# Comparison of permeable cell culture inserts for use in culture of a human in vitro air–liquid interface model system

**DOI:** 10.14814/phy2.15921

**Published:** 2024-02-01

**Authors:** Stephanie A. Brocke, Adam M. Speen, Syed Masood, Cameron P. Worden, Ilona Jaspers

**Affiliations:** ^1^ Curriculum in Toxicology and Environmental Medicine University of North Carolina Chapel Hill North Carolina USA; ^2^ Center for Environmental Medicine, Asthma, and Lung Biology University of North Carolina Chapel Hill North Carolina USA; ^3^ Department of Otolaryngology, Head and Neck Surgery University of North Carolina Chapel Hill North Carolina USA

**Keywords:** air–liquid interface, airway epithelial cells, permeable inserts, primary cell culture

## Abstract

In this study, we compared 12 mm cell culture inserts with permeable polyester membranes (0.4 μm pores) from two different manufacturers: CELLTREAT® and Corning®. Physical dimensions and masses of the inserts were found to be very similar between the two brands, with CELLTREAT® inserts having a slightly smaller diameter and growth area (11.91 mm; 1.11 cm^2^) compared to Corning® Transwells® (12 mm; 1.13 cm^2^). We compared cell differentiation outcomes of human nasal epithelial cells (HNECs) at air–liquid interface grown on inserts from the two different manufacturers, including trans‐epithelial electrical resistance, ciliary beat frequency, ciliated area, and gene expression. HNECs from three male donors were used for all endpoints. No statistically significant differences were observed between paired cultures grown on different brands of insert. In conclusion, these inserts are comparable for use with airway epithelial cell model systems and likely do not impact cellular differentiation or cell culture quality.

## 1 INTRODUCTION

In recent years, global supply chain issues have disrupted access to certain biomedical supplies and reagents and required research groups to explore alternative approaches, even reusing tissue culture supplies (Kato et al., 2021). Shortages in key product availability and the need to use alternate brands of laboratory supplies have the potential to introduce additional confounding variables in experimental design and increase uncertainty about comparability of results obtained within the same lab. In the present study, we sought to compare various properties of two different brands of permeable cell culture inserts that appeared very similar based on manufacturers' descriptions; Corning® Transwell® inserts and CELLTREAT® Permeable Cell Culture Inserts. Aspects of insert size (height, diameter, growth area, etc.) were compared between the two brands. Additionally, we validated usage of the two brands of insert for culture of human nasal epithelial cells (HNECs) grown at air–liquid interface (ALI) using a variety of functional endpoints, including trans‐epithelial electrical resistance (TEER), ciliary beat frequency, and gene expression.

## 2 MATERIALS AND METHODS

**FIGURE 1:**
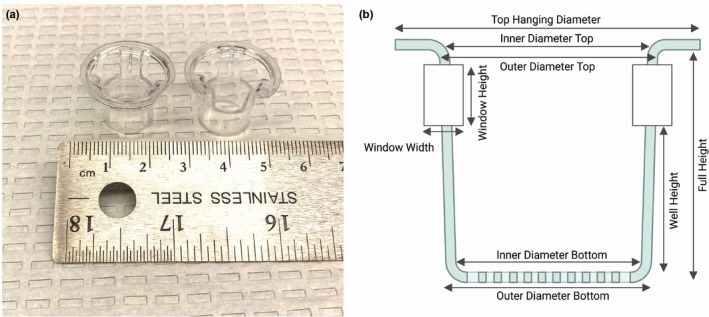
(a) CELLTREAT® (left) and Corning® Transwell® (right) inserts. (b) A diagram of a generic permeable cell culture insert indicating where the various measurements in Table 1 were taken. Image generated with BioRender.com.

Two brands of 12‐well inserts were compared in this study: CELLTREAT® Permeable Cell Culture Inserts with a 0.4 μm pore size‐polyethylene terephthalate (PET) membrane (Pepperell, MA, USA, Product #230621), and Corning® Transwell® inserts with a 0.4 μm pore size‐PET membrane (Corning, NY, USA, Product #3460), shown in Figure 1a. Replicate dimensional measurements were taken on each of three separate inserts of both brands using electronic digital calipers, and replicate masses of individual inserts were obtained. A diagram of the measurements taken is shown in Figure 1b. Additionally, the membranes of both brands of insert were imaged by digital light microscopy using an EVOS FL Auto 2 (Thermo Scientific, Waltham, MA, USA) with a 20× air objective.

Outcomes of cell growth and differentiation using primary HNECs grown at ALI on the two brands of permeable inserts were compared. All HNECs used were obtained from healthy male adult donors who provided written consent per an Institutional Review Board‐approved protocol. Tissue harvest and culture using PneumaCult medium (StemCell Technologies, Inc., Vancouver, BC, Canada) were performed as previously described (Brocke et al., 2022; Müller et al., 2013). To assess rate of HNEC attachment after seeding, live cell imaging was conducted in a time series. Both brands of insert were first collagen coated using type IV human collagen (Advanced Biomatrix, Carlsbad, CA, USA, #5022). Cells from one male donor (*N* = 1) were seeded at 330,000 cells/insert in 200 μL of apical medium on each of three replicate inserts for both brands. Beginning at 1 h post‐seeding, two selected points on each insert were imaged at 30‐min intervals for 10 h on a Nikon A1R‐HD25 confocal imaging system using transmitted light as 10× air objective. Imaging was conducted in a live cell imaging chamber which was maintained at 37°C, 5% CO_2_, and >80% relative humidity. Images were acquired using a Zyla 5.5 sCMOS camera (Andor Technology, Belfast, United Kingdom). Microscopy images were visualized with the NIS‐Elements AR software (Nikon Instruments Corporation, Melville, NY, USA). For the TEER, ciliated area, ciliary beat frequency, and gene expression measurements, HNECs were obtained from *N* = 3 healthy male adults per an Institutional Review Board‐approved protocol (Brocke et al., 2022; Müller et al., 2013). Cells from separate donors were seeded on three inserts of each brand at 280,000 cells/insert and allowed to expand to confluency while submerged. At that point, cultures were switched to ALI conditions with basolateral PneumaCult ALI Medium (StemCell Technologies, Inc.). Subsequently, three times per week basolateral medium was changed and cultures were apically washed with Hank's Balanced Salt Solution, with CaCl_2_ and MgCl_2_ (HBSS++). Endpoints were measured after >30 days of differentiation.

The TEER of each culture was measured using an Epithelial Voltohmmeter 2 (World Precision Instruments, Sarasota, FL, USA). First, 400 μL of warmed HBSS++ was added to the apical surfaces of replicate wells from each donor on both brands of insert, and cells were returned to the incubator for 30 min of equilibration. Following an additional 10‐min equilibration at room temperature, TEER measurements were obtained with STX2 electrodes (World Precision Instruments). TEER of empty inserts (with HBSS++ on the apical side and PneumaCult ALI medium on the basolateral side) was also measured.

To evaluate differences in ciliation, we measured the percent area with active cilia and ciliary beat frequency between cultures grown on the different brands of insert. Immediately before imaging, cultures were washed apically with HBSS++ to remove excess mucus. Experiments were performed at room temperature. Imaging was performed using a Nikon Diaphot inverted microscope, and motion was recorded using a digital camera (Basler AG, Ahrensburg, Germany). Percentage of active area and ciliary beat frequency were measured in four separate fields of view (10× magnification) in three replicate cultures per donor/brand combination and analyzed using SAVA software (Ammons Engineering, Clio, MI, USA) (Sisson et al., 2003).

Finally, transcript‐level expression of six genes related to airway epithelial function was compared in matched cultures grown on each brand of insert by RT‐PCR using Taqman primers and probes, more detail provided in Brocke et al. (2022). Cell lysates from technical replicate wells were pooled for RNA extraction and PCR.

## 3 RESULTS

Physical dimensions of the two brands of inserts are reported in Table 1. This information is provided because diameter and surface area measurements of the permeable membrane of CELLTREAT® inserts are not provided by the manufacturer. Additionally, differences in dimensions of the insert brands may be important for some experimental applications requiring specialized culture conditions or exposure systems. Importantly, the growth area of CELLTREAT® inserts was 111.41 mm^2^ (average diameter 11.91 ± 0.039 mm), which is slightly smaller than the 113.06 mm^2^ (average diameter 12 ± 0.046 mm) growth area of Corning® Transwell® inserts. The well depth of CELLTREAT® inserts was 8.88 ± 0.016 mm while the depth of Corning® Transwell® inserts was 9.09 ± 0.021 mm.

**TABLE 1:** Physical dimensions of both brands (Corning® Transwells® and CELLTREAT®) of 12‐well permeable cell culture inserts with 0.4 μm polyethylene terephthalate membranes.

Measurement (mm)	Corning® Transwell®					CELLTREAT®					Difference
	Insert 1	Insert 2	Insert 3	Mean	Standard deviation	Insert 1	Insert 2	Insert 3	Mean	Standard deviation	(Corning® – CELLTREAT®)
Outer diameter top	14.97	15.05	14.97	15.00	0.0385	14.83	14.84	14.82	14.83	0.0096	0.17
Inner diameter top	12.50	12.44	12.48	12.48	0.0264	12.26	12.44	12.36	12.35	0.0723	0.12
Outer diameter bottom	14.47	14.51	14.54	14.51	0.0286	14.54	14.51	14.57	14.54	0.0233	−0.03
Inner diameter bottom	12.01	11.94	12.05	12.00	0.0457	11.88	11.97	11.89	11.91	0.0385	0.09
Full height	17.27	17.30	17.34	17.30	0.0314	16.13	16.42	16.27	16.27	0.1184	1.03
Well height	9.07	9.08	9.12	9.09	0.0208	8.89	8.88	8.86	8.88	0.0155	0.21
Window width	7.36	7.29	7.31	7.32	0.0279	8.21	8.51	8.45	8.39	0.1291	−1.07
Window height	6.01	6.19	6.09	6.10	0.0708	5.96	5.43	5.62	5.67	0.2163	0.43
Top hanging diameter	25.10	25.18	25.18	25.15	0.0401	24.98	25.03	25.05	25.02	0.0288	0.13
Mass (g)	0.89	0.88	0.89	0.89	0.0009	0.86	0.85	0.85	0.85	0.0035	0.03

*Note*: Three replicate measurements were taken on each of three separate inserts of each brand.

**FIGURE 2:**
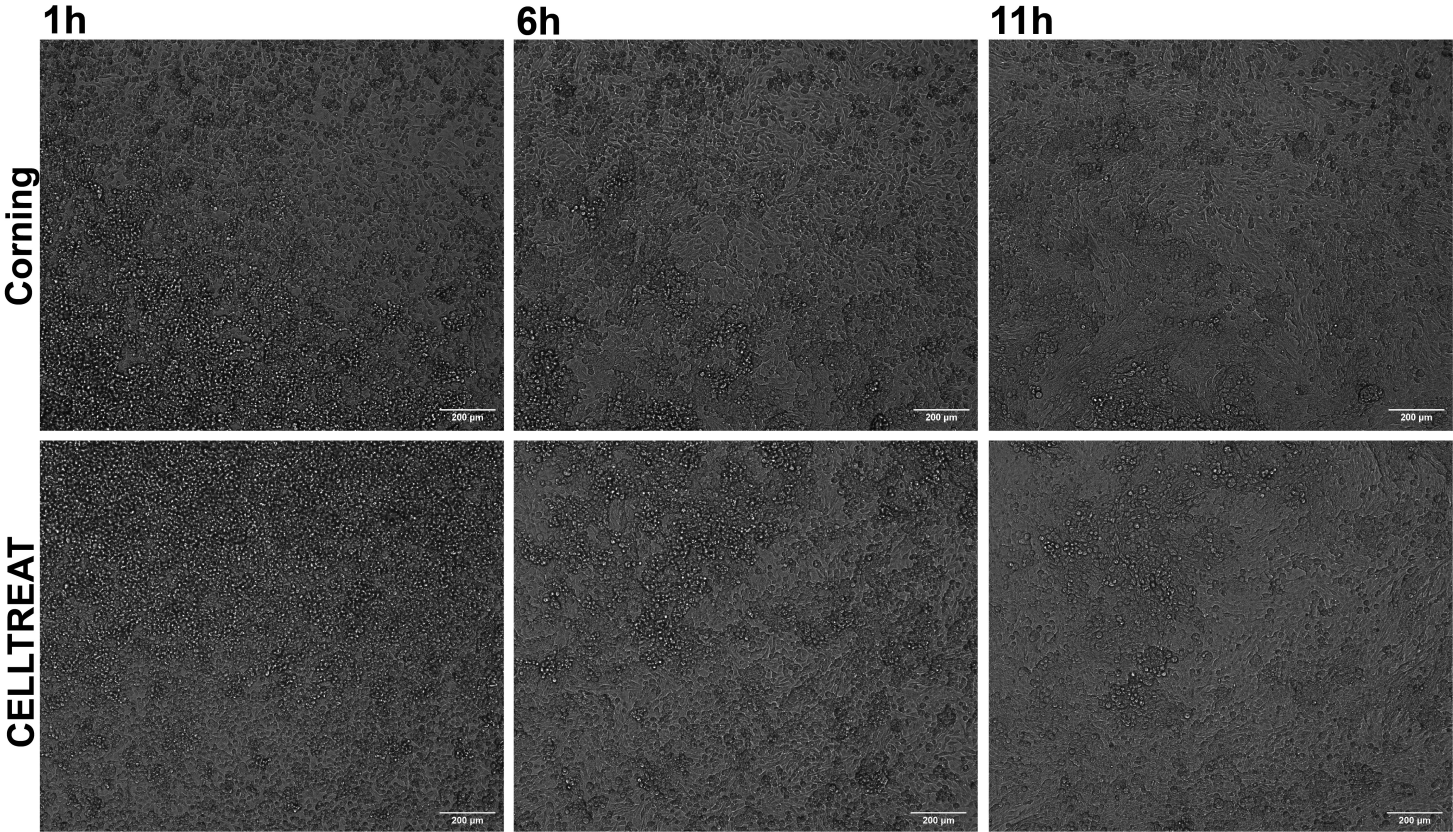
Representative transmitted light images of HNECs from one donor at 1, 6, and 11 h post‐seeding on permeable Corning® (top) and CELLTREAT® (bottom) inserts.

Upon magnification, the appearance of the porous surface of the empty inserts differed by brand (Figure S1). Thus, cell attachment to both brands of insert was assessed in HNECs for 11 h post‐seeding on the inserts. Three replicate inserts were imaged per brand of insert, and representative images at 1, 6, and 11 h are shown in Figure 2. At 1 h post‐seeding, many cells are round in shape and are in focus just above the membrane. By 11 h post‐seeding, the cells are oblong and spread in a monolayer on the membrane surface, indicating uniform adherence. Based on visual inspection, there appears to be no difference in cell attachment by insert brand, with most cells attached to the permeable membranes by 11 h post‐seeding. Representative time‐lapse videos for each insert brand are also provided in the Videos S1 and S2.

**FIGURE 3:**
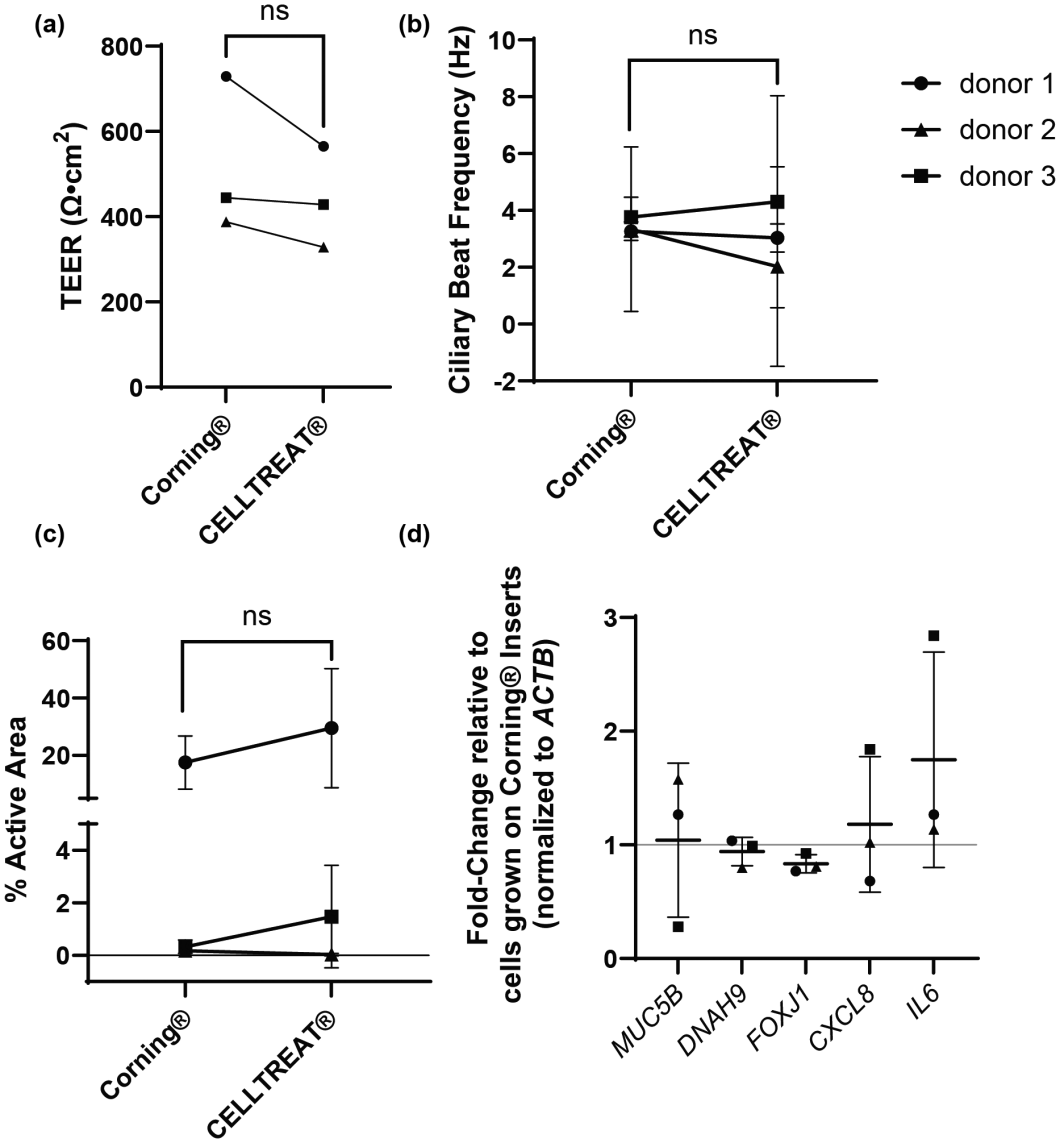
Insert brand did not impact functional endpoints in HNECs (*N* = 3 donors). Across all panels, the same donor is indicated by the shape of the data point (circle, triangle, or square). (a) Blank‐corrected TEER was measured on three replicate wells of each donor (*N* = 3) on both brands of insert. Analyzed by paired *t*‐test, *p* ≤ 0.05 with GraphPad Prism v 9.5.0. (b) Ciliary beat frequency and (c) percentage active area (a measure of culture area covered by motile cilia) was measured in three replicate wells from each donor on both brands of insert and analyzed by SAVA. Statistical testing was done in GraphPad Prism v. 9.5.0 by paired *t*‐test, *p* ≤ 0.05. (d) RT‐PCR was performed on pooled replicate cell lysates from each donor grown on both brands of insert. Fold‐change of mRNA from cells grown on CELLTREAT® inserts, relative to cells grown on Transwells®, and normalized to *ACTB* is shown. Analyzed by one‐sample *t*‐test, *p* ≤ 0.05, in GraphPad Prism v 9.5.0. TEER, trans‐epithelial electrical resistance.

TEER was measured to compare tight‐junction formation in HNEC monolayers grown on the two brands of insert. In the empty inserts, there was a statistically significant (*p* < 0.0001) difference in TEER between the two brands, with CELLTREAT® having an average TEER value of 119.5 ± 6.1 Ω cm^2^ and an average of 177.9 ± 1.2 Ω cm^2^ for Corning®. However, no statistically significant difference in blank‐corrected TEER was observed between cultures grown on the two different insert brands (paired *t*‐test, *p* ≤ 0.05), shown in Figure 3a.

Degree of ciliation and beat frequency of motile cilia were next measured in the differentiated HNEC cultures grown on both insert brands. Donor averages with standard deviation are shown in Figure 3b,c. No statistically significant differences between insert brands were observed for either endpoint (paired *t*‐test, *p* ≤ 0.05). Additionally, there were no product brand differences in time to culture differentiation (i.e., first observation of motile cilia) within the donors (data not shown).

Expression of genes involved in mucin production (*MUC5B*, *MUC5AC*), motile cilia (*DNAH9*, *FOXJ1*), and inflammatory markers (*CXCL8*, *IL6*) was not affected by insert type (one‐sample *t*‐test, *p* ≤ 0.05). Of note, *MUC5AC* mRNA was not detectable in any cultures using the methods described here.

## 4 DISCUSSION

Constraints in the global supply chain, shortages of commonly used research supplies, and availability of alternative products at sometimes lower cost have introduced a variable potentially making comparison of results obtained in the same laboratory or across research groups difficult. Corning® Transwell® inserts exemplify one such product which has been used by numerous groups across the globe to grow and differentiate different cell types and organotypic cultures. In the aftermath of the COVID‐19 pandemic, we and others (Kato et al., 2021) sought to find an alternative to the widely used Transwell® for our ALI airway epithelial cell culture model systems.

CELLTREAT® permeable cell culture inserts were similar to Corning® Transwells® in overall appearance (Figure 1a), dimension, and mass (Table 1). The main physical difference between brands is the presence of a larger window around the top edge of the Corning® inserts which is absent in CELLTREAT® inserts. The growth area of the CELLTREAT® insert was slightly smaller than that of the Transwell®, which should be considered for studies requiring dosage per unit area. Although the rate of cell attachment to both brands of insert was comparable (Figure 2), the physical appearance of the permeable surface of each brand of insert was disparate (Figure S1). Differences in pore density could be an important parameter for studies assessing transmembrane release or migration of submicron materials, thus requiring further studies comparing pore density between the brands.

Primary HNECs differentiated at ALI provide a useful model system for investigating toxicant‐induced effects on the airway mucosa and are a model that has been extensively used in many different studies (Charles et al., 2019; Jaspers et al., 2005; Keegan & Brewington, 2021; Takizawa, 2004; Yoo et al., 2003). Important characteristics of differentiated airway epithelial cell cultures include formation of tight junctions, presence of motile cilia, and production of mucins (Yoo et al., 2003). Measurement of TEER serves as an indicator of monolayer integrity and tight‐junction formation in cell cultures (Srinivasan et al., 2015). No statistically significant difference in TEER was observed between cultures grown on the two brands of insert, indicating similar formation and maintenance of barrier integrity between cells grown on the two different brands of inserts. Similarly, the culture area covered with motile cilia and ciliary beat frequency, markers of differentiation and epithelial cell function, also did not differ between cultures grown on the two insert brands. In the present study, cells from two of the three donors used had much lower ciliated area than the other, which hindered accurate comparison of ciliary characteristics due to the experimental need to keep microscope and light settings consistent for all donors. While this is a limitation of our study, it reflects the observation that inter‐donor variation in overall ciliation of the culture was larger than within‐donor experimental variation between insert brands. However, the cultures grown on CELLTREAT® inserts demonstrated greater variability in both measurements, suggesting potential intra‐product inconsistencies.

Expression levels of many genes are known to increase during differentiation of the mucociliary epithelium (Brekman et al., 2014). As mentioned above, secretion of mucins and presence of motile cilia are markers of differentiation and insert brand had no effect on mRNA expression of genes encoding these markers. Furthermore, expression levels of two pro‐inflammatory mediators, CXCL8 and IL‐6, which are indicators of culture stress and damage, did not differ between cultures grown on different inserts. Together, these data indicate that baseline gene expression levels are similar between HNECs grown on Corning® Transwell® and CELLTREAT® inserts.

## 5 CONCLUSION

In summary, these data suggest that CELLTREAT® inserts are comparable to Corning® Transwell® inserts, resulting in differentiated human airway epithelial cell cultures with similar functional characteristics and baseline gene expression patterns. Based on these data, it seems unlikely that using CELLTREAT® or Corning® Transwell® inserts for culture of human airway epithelial cells would impact cell differentiation or experimental outcomes. In addition to the cell culture inserts examined here, there are others currently available (Fulcher et al., 2005) with comparable features and characteristics. Several manufacturers offer inserts with permeable membranes in a variety of polymers including polycarbonate, PET, and polytetrafluoroethylene. The effect of membrane polymer type on cell culture outcomes is currently understudied. While the data summarized here focused on two different brands of cell culture inserts with PET membranes, similar experiments should be done to validate other model systems and cell types commonly grown on permeable inserts using appropriate functional endpoints.

## AUTHOR CONTRIBUTIONS

SAB, AMS, and IJ were involved in conception and study design. SAB, AMS, SM, and CPW executed experiments and processed data. SAB analyzed data and created figures. SAB and IJ contributed to writing the manuscript. SAB, AMS, SM, CPW, and IJ provided editorial feedback on the manuscript.

## FUNDING INFORMATION

Stephanie A. Brocke and Ilona Jaspers were supported by R01ES031173 from the NIEHS. Stephanie A. Brocke was also supported by T32ES007126 from the NIEHS. Cameron Worden was supported by T32DC005360 from NIDCD. This work was partially funded by the National Institutes of Health. Syed Masood was supported by US EPA Cooperative Agreement CR84033801.

## CONFLICT OF INTEREST STATEMENT

The authors have no affiliations or conflicts of interest to disclose.

## ETHICS STATEMENT

The research was conducted ethically in accordance with the declaration of Helsinki. The institutional Revew Board of the University of North Carolina approved the collection of human nasal epithelial cells and written consent was obatined from all study volunteers.

## Supporting information


Figure S1.

Video S1.

Video S2.

## Data Availability

The data that support the findings of this study are available from the corresponding author upon reasonable request.
