# Supplementary figures and images for: Comparison of permeable cell culture inserts for use in culture of a human in vitro air–liquid interface model system
Source: Physiol Rep. 2024 Feb 1;12(3):e15921. doi: 10.14814/phy2.15921 (PMC10834316; doi:10.14814/phy2.15921)

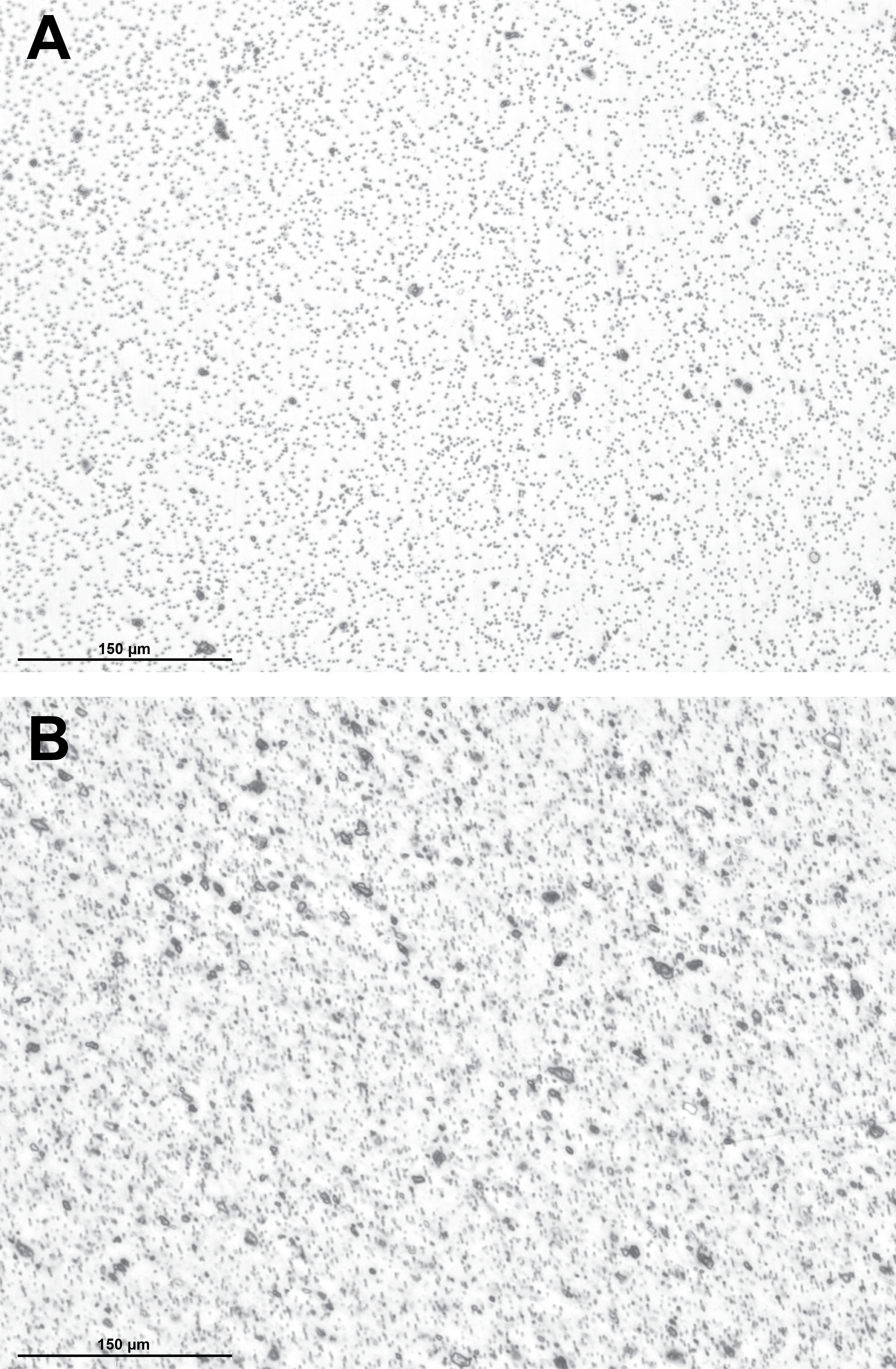

Supplement: Supplementary file 1 — Figure S1. [file PHY2-12-e15921-s003.png]
